# The potential anti-seizure effects of Astaxanthin-loaded nanostructured lipid carriers in rat model of status epilepticus

**DOI:** 10.3389/fnmol.2025.1613893

**Published:** 2025-06-26

**Authors:** Sherien E. Khalaf, Mohammad Al Masri, Ghaleb Oriquat, Maher A. Kamel, Nagwa M. Assem, Suzan M. Abdel-Tawab, Samar S. Elblehi, Shimaa A. Mahmoud

**Affiliations:** ^1^Department of Biochemistry, Medical Research Institute, Alexandria University, Alexandria, Egypt; ^2^Department Audiology and Speech Pathology, Al-Ahlyyia Amman University, Amman, Jordan; ^3^Department of Medical Laboratory Sciences, Faculty of Allied Medical Sciences, Al-Ahliyya Amman University, Amman, Jordan; ^4^Pharos University in Alexandria, Alexandria, Egypt; ^5^Department of Pathology, Faculty of Veterinary Medicine, Alexandria University, Alexandria, Egypt

**Keywords:** Astaxanthin, carbamazepine, epilepsy, GABA, GABA_A_ receptors, nanostructured lipid carriers

## Abstract

**Introduction:**

Epilepsy is a common neurological disorder; seizures and hyperexcitability are its defining features in the central nervous system (CNS). The condition known as status epilepticus (SE) can be fatal, as it involves seizures occurring. Epilepsy is typically treated with antiepileptic drugs (AEDs) like carbamazepine (CBZ). The present study aimed to establish a rat model of SE-like disease using the LiCl-pilocarpine and then utilize these rat models to evaluate the therapeutic potential of AST and/or CBZ in a solution form or loaded on NLCs via intranasal administration. Additionally, to investigate the potential molecular targets of AST and AST + CBZ-nanoformulations.

**Methods:**

After the treatment was completed, the rats underwent behavioral tests, including the rotarod and Morris Water Maze (MWM). They are then sacrificed and their brains were dissected to obtain the cerebral cortex and hippocampus for the assessment of neurotransmitters such as gamma-aminobutyric acid (GABA), serotonin, and dopamine; gene expression of GABA type A (GABAA) receptors subunits and gephyrin; indicators of inflammation like nuclear factor kappa-light-chain-enhancer of activated B cells (NF-κB) and High-Mobility Group Box 1 (HMGB1); antioxidant markers, including nuclear factor transcription factor E2-related factor 2 (Nrf2) and hem oxygenase-1 (HO-1).

**Results:**

The rats treated with the combination of AST and CBZ in nano-formulations seeing the best results.

**Discussion:**

Astaxanthin (AST) may reduce epilepsy-induced oxidative stress and neuronal cell death in the brain. Nano lipid carriers (NLCs) serve as better drug delivery carriers for lipophilic drugs such as CBZ and AST. AST exhibited potential anti-epileptic effects on its own, particularly as NLC-nanoformulations and when combined with conventional AEDs (CBZ).

## 1 Introduction

Status epilepticus (SE) is the most prevalent neurological emergency condition, characterized by ongoing or recurring seizures lasting at least 5 min. Along with an inadequate return of consciousness in between seizures. SE affects around 61 out of every 100,000 individuals each year, resulting in serious, lasting effects and a high death rate. Individuals with SE experience continuous seizures, making the condition life-threatening (Trinka et al., 2015).

Epileptic seizures can develop due to a relative imbalance between inhibitory neurotransmitters, especially the gamma-aminobutyric acid (GABA), and excitatory neurotransmitters (e.g., glutamate) (Sarlo and Holton, 2021). GABA binds to two types of receptors: GABA_A_ and GABA_B_. GABA_A_R subunits are subdivided into seven classes—*α* (1–6), *β* (1–3), *γ* (1–3), *δ*, *ε* (1–3), *θ*, and *π*—based on sequence homology. The majority of GABA_A_Rs consist of the 2α, 2β, and 1γ (or 1δ) subunits. The proper formation and stabilization of inhibitory GABA_A_Rs depend on a specific protein called gephyrin, which serves as a scaffold molecule that attaches glycine and GABA_A_ receptors to the subsynaptic membrane at inhibitory synapses (Pizzarelli et al., 2020).

Nuclear factor kappa-light-chain-enhancer of activated B cells (NF-κB) and high-mobility Group box 1 (HMGB1) are the primary mediators of neuroinflammation that are involved in epilepsy pathogenesis. Neurons and glial cells actively produce HMGB1 and bind to the Toll-like receptor 4 (TLR4), causing nuclear factor kappa-light-chain-enhancer of activated B cells (NF-κB) to trigger the production of proinflammatory cytokines (Iori, 2018). Neuroinflammation is linked to the induction of oxidative stress due to the increased generation of reactive oxygen species (ROS) and/or diminished antioxidant systems, such as nuclear factor erythroid 2 regulated factor 2 (Nrf2), a transcription factor that regulates the expression of most antioxidant systems. This leads to brain damage that contributes to neuronal death and the development of epilepsy (Borowicz-Reutt and Czuczwar, 2020).

A substantial treatment gap remains despite the dramatic changes in the epilepsy treatment landscape over the past decades. Epilepsy is usually controlled by old antiepileptic drugs (AEDs) such as carbamazepine (CBZ) and valproic acid (VPA), and currently, a new generation of AEDs is available. However, the older AEDs are still widely prescribed due to their well-established efficacy and safety profiles. CBZ is a lipophilic drug that exerts its pharmacodynamic action by blocking voltage-activated sodium channels, thereby stabilizing overexcited neural membranes and inhibiting seizure activity in epilepsy. It belongs to the WHO model list of essential medicines and is still considered the standard of care and the most prescribed initial monotherapy for epilepsy, despite the emergence of new AEDs (Beydoun et al., 2020). CBZ is associated with troublesome adverse events, including unsteadiness, drowsiness, weak coordination, dizziness, and double vision (Cockerell et al., 1995) platelet apoptosis and thrombocytopenia (Xiao et al., 2021). Also, many of the patients showed a degree of resistance to CBZ (Campos et al., 2016). Therefore, alternative drugs, co-treatments, or carrier systems must be explored to replace or enhance the delivery of CBZ to the brain, thereby increasing efficacy and minimizing side effects.

Previously, we demonstrated the effective therapeutic effects of Astaxanthin (AST) administered either as a solution or loaded on the nanolipid carrier (NLC) in a rat model of Alzheimer’s disease via the intranasal route. This method serves as a non-invasive way to bypass the blood–brain barrier and deliver drugs directly to the brain (Shehata et al., 2023). Astaxanthin (AST) is second-generation antioxidant and one of the most potent natural compounds with remarkable antioxidant activity, which can protect tissues from oxidative stress (Lu et al., 2015) and neuroinflammation (Wang et al., 2020). The neuroprotective and therapeutic effects of AST against neurological disorders, such as Alzheimer’s disease, Parkinson’s disease, multiple sclerosis, cerebral ischemia, subarachnoid hemorrhage, traumatic brain injury, spinal cord injury, cognitive impairment, and neuropathic pain, deserve attention due to its ability to cross the blood–brain barrier (Si and Zhu, 2022). Also, AST is known to inhibit platelet aggregation (Deng et al., 2017). All of these effects suggest AST as a promising mono or adjuvant co-therapy of epilepsy.

Given the broad spectrum of neurological effects of AST, we have selected adjunctive use of AST with CBZ in a training animal model of epilepsy, where AST serves as a neuroprotective agent against neuroinflammation and oxidative stress, while CBZ is an effective first-generation antiepileptic drug. The application of lipid nanoparticles like nanostructured lipid carriers (NLC) can protect the encapsulated lipophilic drugs (such as AST and CBZ) from enzymatic degradation, improve solubility, and increase drug bioavailability and stability in the brain (Costa et al., 2021). The present study aimed to evaluate the therapeutic potential of intranasal AST and CBZ mono or combined therapy as solutions or as NLC formulations in rats with epilepsy-like disease (SE-like) using the LiCl-pilocarpine model. Also, to explore the possible molecular targets of these therapies. AST can easily degrade in the presence of light, high temperatures, and oxygen. The expected findings may open a new avenue for developing safe and effective intranasal co-therapy using natural compounds and conventional AEDs to manage SE. This is of great significance and novelty as there have been no studies on the intranasal co-therapy of natural compounds and AEDs in managing SE to date.

## 2 Materials and methods

### 2.1 Formation of carbamazepine (CBZ), Astaxanthin (AST), and (CBZ + AST) loaded nanostructured lipid carriers (NLC)

#### 2.1.1 Materials

Tween 80, oleic acid, and glycerylmonostearate (GMS) were sourced from Lobachemie Fine Chemicals and Laboratory Reagents in India. Carbamazepine (CBZ) was purchased from Sigma–Aldrich (Germany). Astaxanthin (AST) was purchased from Carbosynth (England).

#### 2.1.2 Methods of preparation

Drug–loaded NLCs were prepared by hot high-pressure homogenization (HPH) technique using glycerylmonostearate (GMS) as the solid lipid, oleic acid as the liquid lipid, and Tween 80 as the surfactant. Briefly, the GMS was melted 5°C above its melting point. The liquid lipid was added to the molten lipid and maintained at the same temperature. The concentration of total lipids was 1.5% w/v, and the solid lipid-to-liquid lipid ratio was 90:10. An accurate amount of AST (40 mg) and/or CBZ (10 mg) was added to the molten lipid. The aqueous phase was prepared by dissolving the surfactant (Tween 80, 1.5% w/v) in filtered distilled water and heating it to the same temperature as the lipid phase, which is molten. After 20 min of continuous magnetic stirring (IKA Labortechnik, Staufen, Germany) at 1,000 rpm, the heated aqueous solution was introduced to the molten lipid phase. High-speed homogenization (IKA®T25 digital ultra-TURRAX, Germany) was then conducted for 15 min at 20,000 rpm. To enable the lipid to recrystallize for the creation of NLCs, the generated nanoemulsion was chilled overnight at 4–8°C (Elmowafy et al., 2018; Sun et al., 2017).

#### 2.1.3 Characterization of the manufactured NLCs containing drugs

The Malvern Zetasizer was used to determine the mean particle size (PS), polydispersity index (PDI), and zeta potential (ZP) of the prepared drug-loaded NLCs (Malvern Instruments, UK) after diluting the samples with filtered distilled water. The value is the mean of three replicates ± standard deviation (SD) (Tamjidi et al., 2014).

#### 2.1.4 Stability of the manufactured NLCs containing drugs

The prepared drug-loaded NLCs were subjected to a stability study at 4 and 25°C over a 3-month study period. The samples were withdrawn, evaluated for particle size, and compared with the zero-time data of the freshly prepared drug-loaded NLCs.

#### 2.1.5 Determination of entrapment efficiency (% EE) of the manufactured NLCs containing drugs

The entrapment efficiency (%EE) of the prepared drug-loaded NLCs was determined using the centrifugal ultrafiltration technique. Notably, 5 mL of the prepared formulation was centrifuged using a cooling centrifuge at 10,000 rpm for 20 min at 4°C. The supernatant was collected, filtered, and analyzed to determine the amount of free unloaded drug. High-performance liquid chromatography (HPLC) was used to analyze the AST in the supernatants of AST-NLCS and AST + CBZ-NLC (Agilent Technologies 1,200 Infinity Series, United States), while analysis of CBZ in the supernatants of both CBZ-NLCS and AST + CBZ-NLC was performed using a ultraviolet (UV) electrophoretic method using ultraviolet visible (UV)-Visible spectrophotometer (Shimadzu Corporation, Japan).

The following formula was used to calculate the %EE:

%Entrapment Efficiency (%EE) = (Total amount of drug in 5 mL of drug-loaded NLCs − the amount of free drug in the supernatant) ×100/Total amount of drug in 5 mL of drug-loaded NLCs

### 2.2 *In vivo* study

#### 2.2.1 Animals

A total of 104 healthy adult male albino rats weighing between 150 and 200 g were used in the investigation. They were acquired from the animal house of the Medical Research Institute at Alexandria University in Egypt. Before and after the trial, all rats had unrestricted access to food and water, a 12:12 light/dark cycle, and consistent environmental circumstances. The Institutional Animal Care and Use Committee (IACUC) at Alexandria University in Egypt conducted all of the experiments in accordance with the National Institutes of Health’s Guide for the Care and Use of Laboratory Animals (NIH Publications No. 8023, revised 1978) (Approval No.: AU0122122321). Additionally, the study complies with the National Research Council’s regulations for the care and use of laboratory animals as well as ARRIVE criteria.

#### 2.2.2 Induction of status epilepticus (SE) in rats using LiCl-pilocarpine

An epileptic model was established using lithium chloride (LiCl) (LOBA Chemie, India) and pilocarpine (Meskinimood et al., 2019). The rats received an intraperitoneal (i.p.) injection of lithium chloride (127.3 mg/kg in 0.9% saline) 24 h before the administration of pilocarpine (García-García et al., 2017). Scopolamine (1 mg/kg in 0.9% saline) (Carbosynth, England) is administered intraperitoneally 30 min before pilocarpine injection to minimize peripheral side effects. Next, 30 mg/kg body weight (b.wt) of pilocarpine, dissolved in 0.9% saline, was administered intraperitoneally to the rats. In the normal group, saline was injected as a replacement for lithium chloride and pilocarpine. Every 30 min, rats were given an intraperitoneal injection of 10 mg/kg pilocarpine if they did not experience a seizure that scored grades IV and V (Deng et al., 2019; Sekhar et al., 2024).

#### 2.2.3 Seizure grading

Seizures were graded using the following classification: I—standing still or wet dog shakes; II—rhythmic nodding and chewing; III—unilateral forelimb clonic seizure; IV—bilateral forelimb clonic and convulsive seizure withstanding; and V—receding, tumbling, with a generalized tonic–clonic seizure. Rats that produced seizures with a persistent state and seizure scores of grade IV–V within 30 min were considered to have successfully triggered status (Ishida et al., 1992).

#### 2.2.4 Experimental design

The adult male albino rats were divided into two main groups, Group I (control group): consisting of 8 normal male rats, and Group II (SE-like rats): epileptic rats (96 rats) were subdivided into 8 groups (12 rats each): epileptic rats were subdivided into eight groups: Group IIA: untreated rats, and 20 μL of the tested formulations were administered intranasally (10 μL in each nostril) to treatment groups (Group IIB to Group IIH) every day for 4 weeks.: Group IIB (NLCs): treated intranasally with NLCs (vehicle), Group IIC (AST-sol.): treated intranasally with 4 mg/kg AST solution (Bhatt et al., 2016), Group IID (CBZ-sol.): treated intranasally with 0.2 mg/kg CBZ solution (Barakat et al., 2006), Group IIE (AST + CBZ-sol.): treated intranasally with a combination of AST (4 mg/kg) and CBZ (0.2 mg/kg), Group IIF (AST-Nano): treated intranasally with AST-NLC (4 mg/kg), Group IIG (CBZ-Nano): treated intranasally (Hanson et al., 2013) with CBZ-NLC (0.2 mg/kg), and Group IIH (AST + CBZ-Nnano): treated intranasally with a combination of AST + CBZ-NLC. For proper intranasal delivery, the rats’ necks were kept parallel to the floor as they were hand-restrained with an altered scruff in the non-dominant hand. The dominant hand was then used to pipette 10 μL of the preparations.

**Figure 1:**
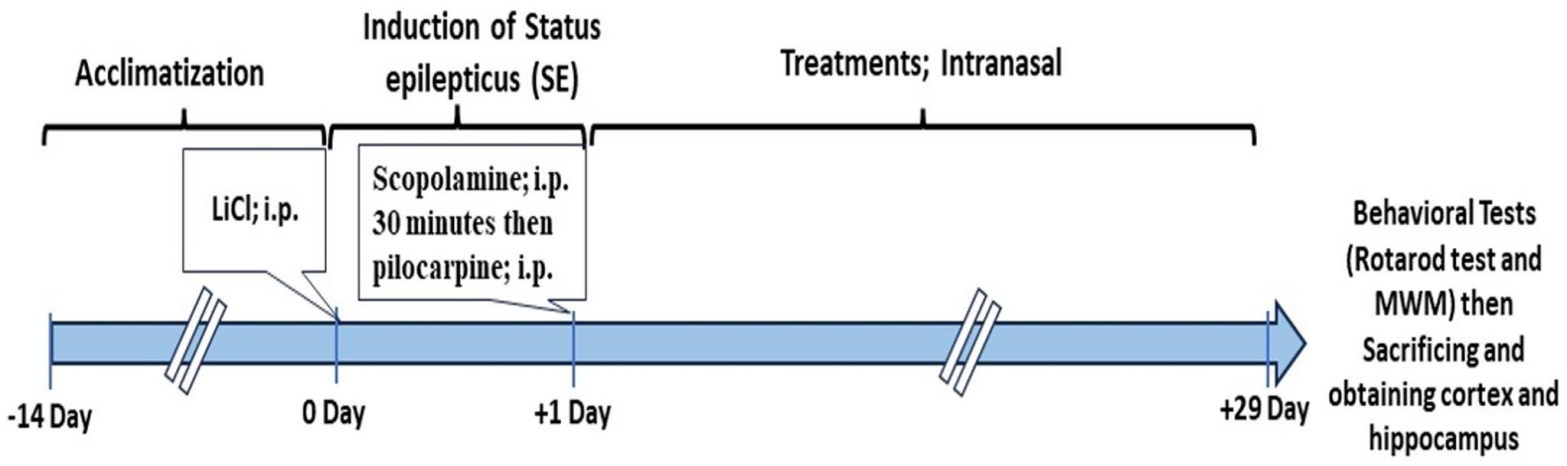
Time-line of the study.

After the treatment period, eight rats of each group were selected from viable rats after the end of the experiments and were subjected to behavioral tests, including the Morris Water Maze (MWM) and rotarod performance test, as described briefly in the “Methods” section. The flowchart explaining the experimental design’s timeline is displayed in Figure 1.

#### 2.2.5 Randomization and blinding procedures

Randomization: The 72 healthy adult male albino rats were randomly assigned to the experimental groups using a computer-generated randomization sequence. Before the induction of status epilepticus (SE), rats were numbered and allocated to either Group I (control, *n* = 8) or Group II (SE-like rats, *n* = 64) using a random number generator (Microsoft Excel, RAND function). Group II was further subdivided into eight subgroups (IIA to IIH, n = 8 per subgroup) through a second randomization step to ensure balanced distribution across treatment conditions: untreated SE-like rats (IIA), NLC vehicle (IIB), AST solution (IIC), CBZ solution (IID), AST + CBZ solution (IIE), AST-NLC (IIF), CBZ-NLC (IIG), and AST + CBZ-NLC (IIH). Randomization was performed by a researcher not involved in the induction or treatment procedures to minimize selection bias. Baseline characteristics, such as body weight, were assessed before randomization to ensure homogeneity across groups, and no significant differences were found [*p* > 0.05, analysis of variance (ANOVA)].

Blinding was implemented at multiple stages to reduce bias during treatment administration, behavioral testing, and data analysis. The personnel administering the intranasal treatments were blinded to the treatment group assignments. Treatment solutions and NLC formulations were prepared and labeled with coded identifiers by an independent researcher, ensuring that the administering team was unaware of whether the rats received vehicle, AST, CBZ, or their combinations in solution or NLC form. During behavioral assessments (rotarod and Morris Water Maze tests), the evaluators were blinded to the group assignments. Similarly, for biochemical, molecular, and histopathological analyses, samples (cortical and hippocampal tissues) were coded to conceal group identity. Data analysis, including statistical comparisons using ANOVA and Pearson correlation, was conducted by a statistician who was blinded to the experimental groups to ensure an impartial interpretation of the results. These procedures were designed to minimize bias and enhance the reliability of the study’s findings, aligning with ethical and scientific standards for animal research.

#### 2.2.6 Euthanasia and sample preparation

After the last day of treatment, eight rats from each group were selected from the viable rats and subjected to behavioral tests (Morris Water Maze and rotarod performance test). Mortality rates were evaluated across all groups. For euthanasia, rats were deeply anesthetized with isoflurane (4–5% induction, 1–2% maintenance) in an anesthesia chamber until loss of consciousness was confirmed by the absence of pedal reflex and corneal response. Subsequently, cervical dislocation was performed by trained personnel to ensure rapid and humane euthanasia, following the National Institutes of Health’s Guide for the Care and Use of Laboratory Animals (NIH Publications No. 8023, revised 1978) and the Institutional Animal Care and Use Committee (IACUC) at Alexandria University (Approval No.: AU0122122321). Cervical dislocation was chosen to avoid chemical contamination of brain tissue, which could interfere with subsequent biochemical, molecular, and histopathological analyses. Post-euthanasia, the brains were dissected out to extract tissues from the cortex and hippocampus, which were then rinsed with saline and homogenized at a 1:10 ratio (0.25 g of tissue in 2.25 mL phosphate buffer saline [PBS]) in PBS with a pH of 7.4 containing 1.0 mM phenylmethanesulfonyl fluoride (PMSF), a serine protease inhibitor. Following homogenization, the homogenates were centrifuged in the centrifuge for 20 min at 4°C at 10,000 *g*. To determine the levels of gamma-aminobutyric acid (GABA), dopamine (DA), serotonin, NF-κB, NRF2, and protein, the supernatants were separated into aliquots and stored at −20°C.

#### 2.2.7 Methods

##### 2.2.7.1 Mortality rate

The mortality rate among the animals was calculated using the following equation:

Mortality rate (%) = (Number of rats that died/total number of rats in the group) × 100.

##### 2.2.7.2 Rotarod performance test

At the end of the treatment period, A rotarod machine was used to assess each rat’s locomotor (motor coordination) abilities. It evaluated the animals’ motor control, balance, and coordination. Following the administration of the therapy, the procedure was carried out. The animals were positioned on the 7 cm diameter rotating rod, which was moving at a steady 20 rpm. Notably, the 90 s spent on the rod was measured (Latency to fall) (Huang et al., 2002).

##### 2.2.7.3 Morris water maze

The Morris water maze (MWM) test was also used to evaluate the cognitive brain functions. Groups of animals underwent spatial reference learning in the MWM test after treatment (Vorhees and Williams, 2006). To assess spatial placement skills, the rat’s swimming courses within 60 s, the time spent in the target quadrant, and the number of swims across the initial platform site were recorded. A stopwatch was used to manually measure the time spent in the correct quadrant and the latency (the time required to reach the concealed platform).

##### 2.2.7.4 ELISA assays

Determination of gamma-aminobutyric acid (GABA), dopamine (DA), serotonin, NF-ĸB, and Nrf2 contents was assayed using rat-specific Enzyme-linked Immunosorbent Assay (ELISA) kits (Chongqing Biospes Co., China) according to the manufacturer’s instructions. A modification method of Lowry et al. was used for the determination of total proteins in the samples (Lowry et al., 1951).

##### 2.2.7.5 Gene expression analysis using real-time PCR

###### 2.2.7.5.1 Total RNA extraction

The total messenger ribonucleic acid (mRNA) was isolated from the cortical and hippocampal tissue using the RNeasy Mini Kit (Qiagen, Germany) according to the manufacturer’s instructions.

###### 2.2.7.5.2 Reverse transcription

The extracted mRNAs were reverse-transcribed using TOPscript™ RT DryMIX (dT18/dN6 plus) kit according to the manufacturer’s instructions (Enzynomics Co., Ltd., Korea, category number: RT220).

###### 2.2.7.5.3 PCR quantification

Relative quantification of GABA_A_ receptor subunits (α1, α2, α5, β1, β2, γ2, and γ3), gephyrin, HMGB1, NRF2, HO-1, and 18S rRNA gene expression was performed using Topreal™ qPCR 2X preMIX (*SYBR Green with low Rox*) (enzynomics, Korea) and using the specific primer pair for each gene (Table 1). The Bio-Rad CFX Maestro version 2.3 (Bio-Rad, Inc., United States) was used to collect the data. The PCR amplification conditions were as follows: initial denaturation at 95°C for 5 min, followed by 45 cycles of amplification, consisting of denaturation at 94°C for 20 s, annealing at 55°C for 20 s, and extension at 70°C for 15 s. The relative expression of P GABA_A_ receptor subunits (α1, α2, α5, β1, β2, γ2, and γ3), gephyrin, HMGB1, NRF2, and HO-1 were quantified in the same sample relative to the reference gene (18S rRNA) by normalizing the target gene’s threshold cycles (Ct) values to those of 18S rRNA. The fold change of expression of each gene relative to the control samples was calculated using the Livak 2^-ΔΔCt^ method (Livak and Schmittgen, 2001).

**Table 1:** Primer sets of gamma-aminobutyric acid type A (GABA_A_) receptors (GABA_A_α1, GABA_A_α2, GABA_A_α5, GABA_A_β1, GABA_A_β2, GABA_A_γ2, and GABA_A_γ3), gephyrin, high mobility group box 1 (HMGB1), nuclear factor erythroid 2 related factor 2 (NRF2), heme oxygenase-1 (HO-1), and 18 s.

Gene name	Accession number	Primer sequence	
GABA_A_a1	NM_183326	F:	5’-TGACAGTCATTCTCTCCCAAGTC-3’
		R:	5’-TCAGAACGGTCGTCACTCC-3’
GABA_A_a2	NM_001135779	F:	5’-GACTGTCATTCTCTCCCAAGTGT-3’
		R:	5’-TCATTGTCAAAACAGTTGTTACTCC-3’
GABA_A_a5	NM_017069	F:	5’-CCTACTTGCCATGTATCATGACTG-3’
		R:	5’-GGTCATGGTGAGAACAGTGGT-3’
GABA_A_β1	NM_012956	F:	5’-CCCTCTGGATGAGCAAAACT-3’
		R:	5’-CCCTCTCCTCCATTCCAGTA-3’
GABA_A_β2	NM_012957	F:	5’-CATCGATATGGTTTCTGAAGTCAA-3’
		R:	5’-GGGATTACATTGTAGGACAGTCTCTT-3’
GABA_A_γ2	NM_183327	F:	5’-ACAGAAAATGACGCTGTGGA-3’
		R:	5’-ATCTGACTTTTGGCTAGTGAAGC-3’
GABA_A_γ3	NM_024370	F:	5’-AGCGAGTGGAGACCAAGC-3’
		R:	5’-CTCTTCCACCCTCCTGGAC-3’
Gephyrin	NM_022865.4	F:	5’-CTGGACCCTCGCCCAGAATA-3’
		R:	5’-CTGTCTTTGGAGGTAGCATCAGCA-3’
HMGB1	NM_012963.2	F:	5’-TGATTAATGAATGAGTTCGGGC-3’
		R:	5’-TGCTCAGGAAACTTGACTGTTT’-3’
NRF2	NM_031789.2	F:	5’-CAAATCCCACCTTGAACACA-3’
		R:	5’-CGACTGACTAATGGCAGCAG-3’
HO-1	NM_012580	F:	5’-CCAGGCAGAGAATGCTGAGTTC-3’
		R:	5’-AAGACTGGGCTCTCCTTGTTGC-3’
18S	NR_046237.2	F:	5’-GGTAACC CGT TGA ACC CCATT-3’
		R:	5’-CAA GCT TAT GAC CCG CAC TT-3’

#### 2.2.8 Histopathological examination and morphometric tissue analysis

##### 2.2.8.1 Histopathological examination

Following necropsy, brain tissue specimens were obtained from different rat groups (*N* = 5/group), rinsed in saline solution, and then placed for at least 24 h in 10% neutral buffered formalin (pH 7.4). Fixed tissue specimens have been processed using the conventional paraffin embedding technique. Brief, paraffin blocks were sectioned at 4.5 μm, dewaxed, and stained with Mayer’s hematoxylin and eosin (H&E) stain. Then the sections were dehydrated with alcohol, cleared with xylene, and sealed. Stained sections were examined by light microscope (Leica, DM500) and photographed using a digital camera (EC3, Leica, Germany).

##### 2.2.8.2 Morphometric tissue analysis

The number of necrotic and degraded cells in the cerebral cortex and hippocampus (CA1) was measured quantitatively using Image J software (Image J 1.47v, National Institutes of Health, Bethesda, MD, USA). At a magnification level of ×400, these morphometric measurements were performed on images of a minimum of five distinct fields per section (one section per animal; five per group). Every morphometric measurement was carried out in a blind manner.

### 2.3 Statistical analysis

The Statistical Package for the Social Sciences (SPSS) version 18.0 software package (Chicago, IL, USA) was used to analyze the data [30]. The data were presented as mean ± SD and analyzed using Pearson’s correlation analysis and ANOVA for group comparison. At *p* < 0.05, the *p-*value was considered significant. When administered in combination, the factorial design test is used to examine any potential interactions between the separate treatments (Hagen, 2003).

## 3 Results

### 3.1 Preparation and characterization of nanoparticles

#### 3.1.1 Particle size, polydispersity index, and zeta potential measurement

The results of PS, PDI, and ZP are presented in Table 2. The prepared drug-loaded Nanostructured Lipid Carriers (NLCs) exhibited particle sizes below 200 nm, deemed suitable for brain targeting via the nasal route. The low polydispersity index (PDI < 0.3) indicated high uniformity in particle size distribution, and high zeta potential (ZP) values suggested good stability. These are consistent with previous studies (Tamjidi et al., 2014; Elmowafy et al., 2018).

**Table 2:** Particle size (PS), polydispersity index (PDI), and zeta potential (ZP) of NLCs, AST-NLCs, CBZ-NLCs, and AST + CBZ-NLCs.

NLC	PS (nm)	PDI	ZP (mV)
NLCs	131.4 ± 2.46	0.21 ± 0.04	−30.2 ± 3.52
AST-NLCs	142.9 ± 4.35	0.224 ± 0.07	**−**30.3 ± 4.67
CBZ-NLCs	155.2 ± 5.82	0.243 ± 0.09	**−**30.1 ± 5.28
AST+CBZ-NLCs	177.2 ± 7.25	0.27 ± 0.06	**−**31.2 ± 5.43

Data were presented using Mean ± SD. SE-like, status epilepticus-like; NLCs, nanostructured lipid carriers; AST, astaxanthin; CBZ, carbamazepine.

#### 3.1.2 Stability study of drug-loaded nanostructured lipid carriers (AST-NLCs, CBZ-NLCs, and AST + CBZ-NLC)

*Following 3 months of storage* at various temperatures (4–8 ± 2°C and 25 ± 2°C/60 ± 5% RH), we measured PS, ZP, and EE. The developed drug-loaded NLCs were more stable at 4–8 ± 2°C than at 25 ± 2°C/60 ± 5% RH because, for the first 3 months, there was no change in the particle size, ZP, or EE of the developed drug-loaded NLCs at 4–8 ± 2°C. However, after 3 months of storage at 25 ± 2°C/60 ± 5% RH, the PS increased, and ZP and EE (of both CBZ and AST) decreased, possibly as a result of particle aggregation at warmer temperatures (Table 3).

**Table 3:** Particle size (PS) of the stored drug-loaded NLCs under different storage conditions (at refrigerator 4°C and room temperature 25°C) after 3 months of storage.

Time		PS (nm)		
		AST-NLC	CBZ-NLC	AST+CBZ-NLC
At zero time		142.9 ± 4.35	155.2 ± 5.82	177.2 ± 7.25
After 3 months	at 4°C	151.2 ± 5.35	165.8 ± 5.68	190.4 ± 5.83
	at 25°C	174.7 ± 6.94	191.7 ± 8.46	± 8.56

Data were presented using Mean ± SD. SE-like, status epilepticus-like; NLC, nanostructured lipid carriers; AST, astaxanthin; CBZ, carbamazepine.

#### 3.1.3 Entrapment efficiency (% EE) of the prepared drug-loaded NLCs

Both drugs, AST and CBZ, showed high EE in the NLCs as AST showed 92.4 ± 3.57% and CBZ showed 88.5 ± 4.62%.

### 3.2 Results of *in vivo* study

#### 3.2.1 Mortality rate

The mortality rate (%) of rats in the different groups is illustrated in Table 4; the highest mortality rate was observed in the SE-like rats, about 33.3% (4 out of 12 rats died). The treatment regimens decrease the mortality among SE-like rats. AST and CBZ combination (solution and nanoform) showed the lowest mortality rate (especially in the rats treated with the combination).

**Table 4:** The mortality rate (%) of the different studied groups.

Groups	Mortality rate (%)
Control	0
SE-like	33.3
NLC	25
AST-Sol	25
CBZ-Sol	25
AST+CBZ-Sol	10
AST-Nano	20
CBZ-Nano	20
AST+CBZ-Nano	10

SE-like, status epilepticus-like; NLC, nanostructured lipid carriers; AST, astaxanthin; CBZ, carbamazepine; Sol, solution; Nano, nanolipid carrier formulation.

#### 3.2.2 Behavioral outcomes

**Figure 2:**
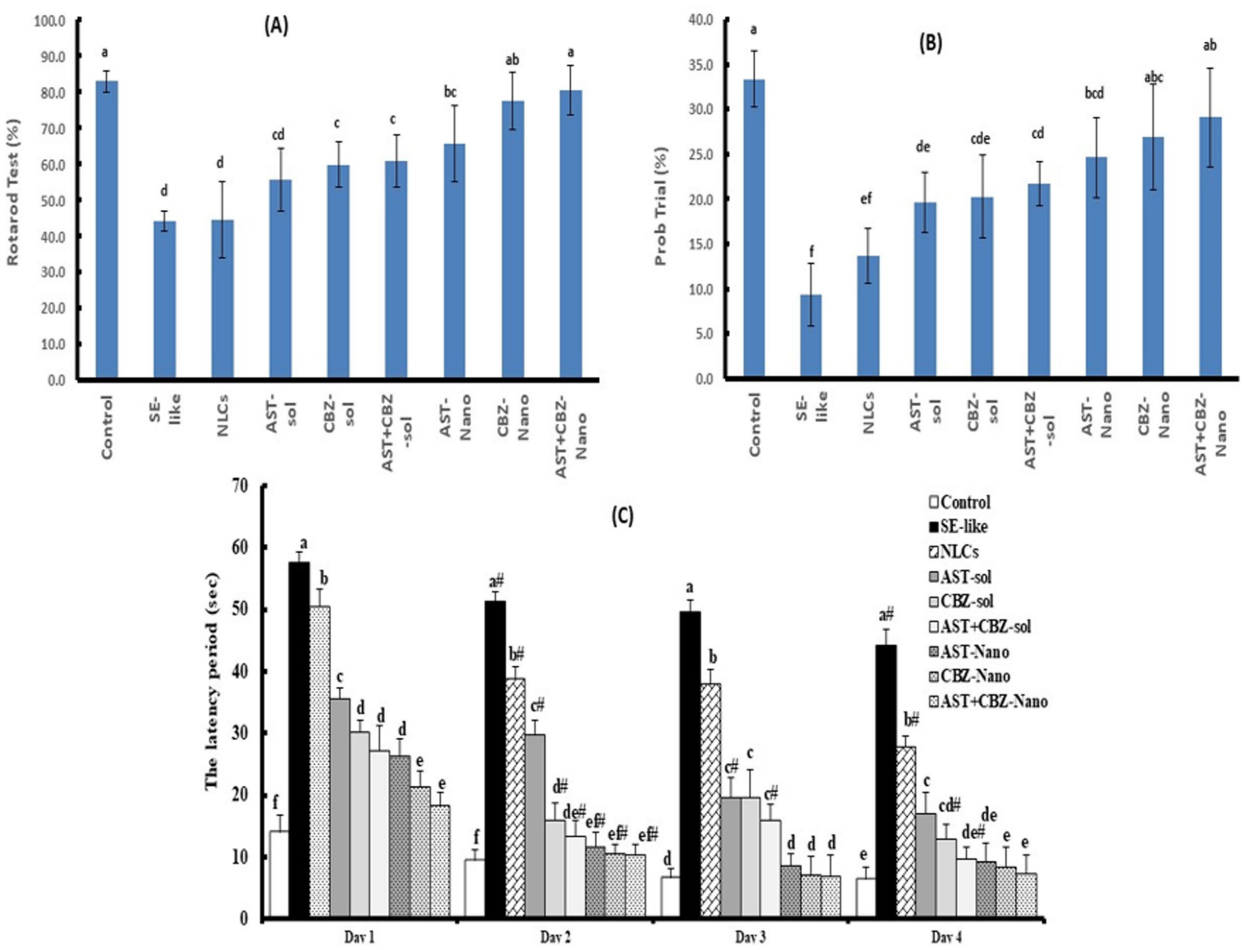
Results of the behavioral tests: Rotarod **(A)** and Morris Water Maze (MWMW) as latency period **(B)**, and the time spent in the correct quadrant during 4-day trials **(C)**. Data presented as Mean ± SD and *n* = 8. Means with common letters are not significantly different, and means with different letters are significantly different by ANOVA followed by Tukey *post-hoc* test, *p* < 0.05. AST, astaxanthin; CBZ, carbamazepine; Nano, nanoparticles; NLCs, nanolipid carriers (vehicle); SE, status epilepticus; sol., solution.

In the rotarod test, SE-like rats (Group IIA) exhibited significantly reduced latency to fall compared to controls, indicating motor impairment (Figure 2A). Treatment with AST-NLC, CBZ-NLC, and AST + CBZ-NLC (Groups IIF–IIH) significantly improved latency (*p* < 0.05 vs. IIA), with AST + CBZ-NLC showing the greatest improvement (Figure 2B). The Morris Water Maze (MWM) revealed that SE-like rats spent less time in the target quadrant and had longer escape latencies vs. the control group (Group I) (Figure 2C). Moreover, rats with epilepsy exhibited altered search patterns in the MWM test, characterized by increased circling and reduced linearity. Groups IIF–IIH showed increased target quadrant time and reduced escape latencies compared to the SE-like group, with AST + CBZ-NLC outperforming monotherapies (*p* < 0.01), suggesting enhanced spatial memory and motor coordination (Figure 2D).

### 3.3 GABAergic pathway

**Figure 3:**
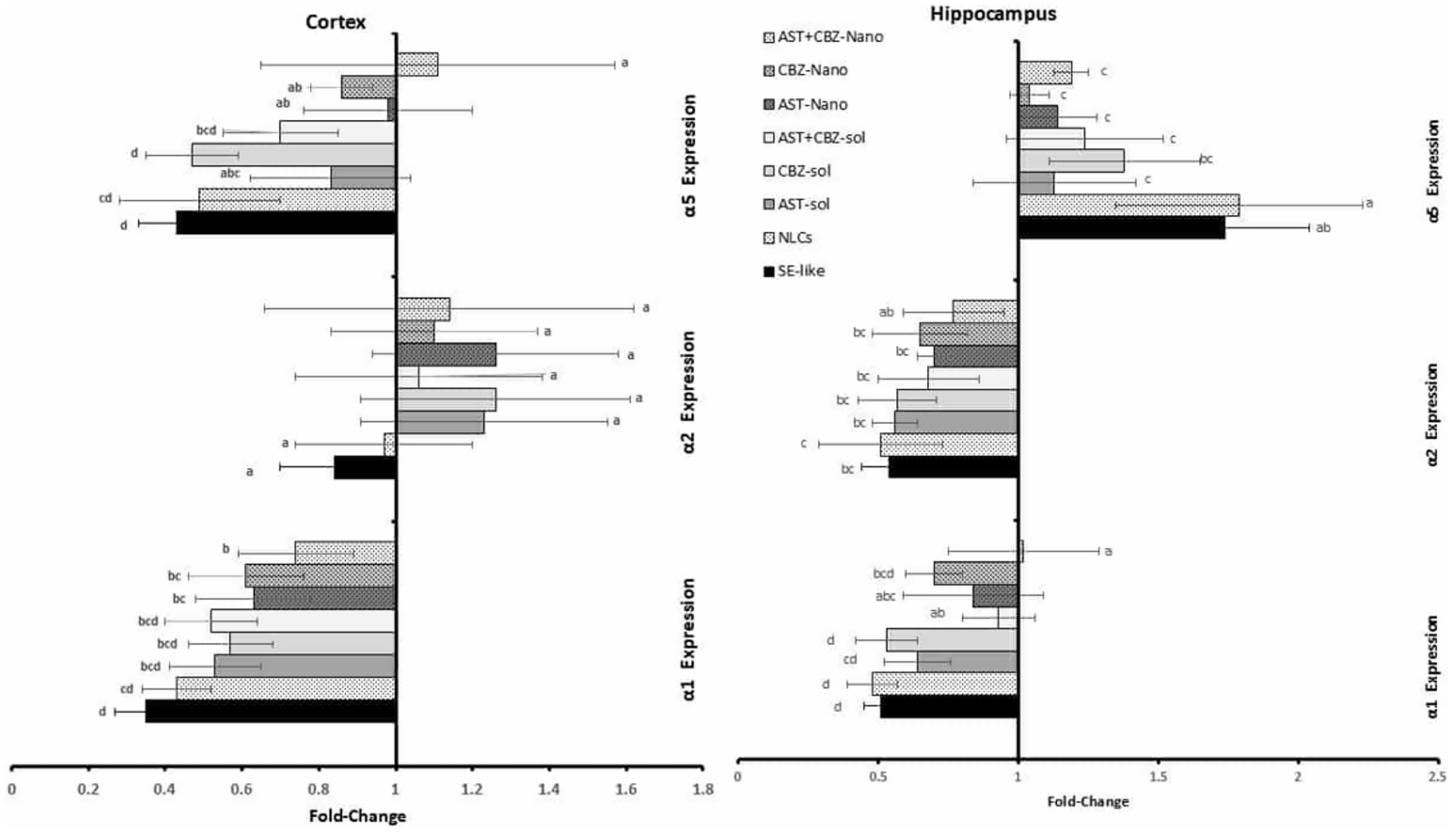
Results of the cortical and hippocampal expression of GABA receptor A (GABA_A_) α1, α2, and α5 subunits of the studied groups. Data presented as Mean±SD and *n* = 8. Means with common letters are not significantly different, and means with different letters are significantly different by ANOVA followed by Tukey *post-hoc* test, *p* < 0.05. AST: astaxanthin, CBZ, carbamazepine; Nano, nanoparticles; NLCs, nano-lipid carriers (vehicle); SE, status epilepticus; sol, solution.

The SE-like rats showed a significant decline in the cortical and hippocampal contents of GABA neurotransmitters compared with the control rats (Table 5). The expression of GABA type A (GABA_A_) receptor subunits in SE-like rats was altered, with significant suppression of cortical gene expression of subunits α1 and α5. In contrast, hippocampal α1 and α2 were suppressed, and α5 was induced (Figure 3). The treatment of SE-like rats with NLC formulations, particularly the AST + CBZ combination, was effective in increasing the expression of suppressed subunits (α1, α5) in the cortex and normalizing altered expressions (α1, α2, α5) in the hippocampus (Figure 3).

**Table 5:** Neurotransmitter levels in the cortex and hippocampus of the studied groups.

Groups		GABA (ng/mg protein)		Serotonin (pg/mg protein)		Dopamine (pg/mg protein)	
		Cortex	Hippocampus	Cortex	Hippocampus	Cortex	Hippocampus
Control		132.2^ab^ ± 11.87	168.6^a^ ± 8.39	4.18^a^ ± 0.37	4.95^a^ ± 0.51	4.73^b^ ± 0.26	7.63^a^ ± 0.56
SE-like rats	Untreated	118.5^c^ ± 2.34	155.5^c^ ± 4.32	1.95^e^ ± 0.38	3.13^cd^ ± 0.66	2.50^d^ ± 0.29	2.24^d^ ± 0.35
	NLCs	119.2^c^ ± 5.01	140.2^d^ ± 11.18	2.06^de^ ± 0.59	2.82^d^ ± 0.16	2.48^d^ ± 0.57	2.05^d^ ± 0.20
	AST-Sol	125.6^bc^ ± 5.42	151.4^c^ ± 6.89	2.72^cd^ ± 0.31	3.46^bcd^ ± 0.32	3.07^cd^ ± 0.38	2.77^bcd^ ± 0.58
	CBZ-Sol	124.3^bc^ ± 2.02	150.8^cd^ ± 6.15	1.96^e^ ± 0.36	3.71^bc^ ± 0.27	3.46^cd^ ± 0.66	2.34^cd^ ± 0.58
	AST+CBZ-Sol	130.8^ab^ ± 1.06	156.9^bc^ ± 2.41	2.05^de^ ± 0.16	3.95^b^ ± 0.72	3.91^bc^ ± 0.59	2.26^d^ ± 0.29
	AST-Nano	132.1^ab^ ± 9.87	166.6^ab^ ± 8.15	4.05^ab^ ± 0.51	3.77^bc^ ± 0.53	3.44^cd^ ± 0.36	3.46^b^ ± 0.40
	CBZ-Nano	127.0^bc^ ± 5.35	159.1^bc^ ± 2.74	3.26^c^ ± 0.43	3.95^b^ ± 0.30	6.27^a^ ± 1.06	3.07^bc^ ± 0.51
	AST+CBZ-Nano	137.3^a^ ± 5.38	168.1^a^ ± 4.44	3.40^bc^ ± 0.60	4.02^b^ ± 0.42	5.86^a^ ± 0.84	3.12^b^ ± 0.55

Data presented as Mean ± SD and *n* = 8. Means in each column with common letters are not significantly different, and means with different letters are significantly different by ANOVA followed by Tukey post hoc test, *p* < 0.05. AST, astaxanthin; CBZ, carbamazepine; Nano, nanoparticles; NLCs, nanolipid carriers (vehicle); SE, status epilepticus; sol, solution.

**Figure 4:**
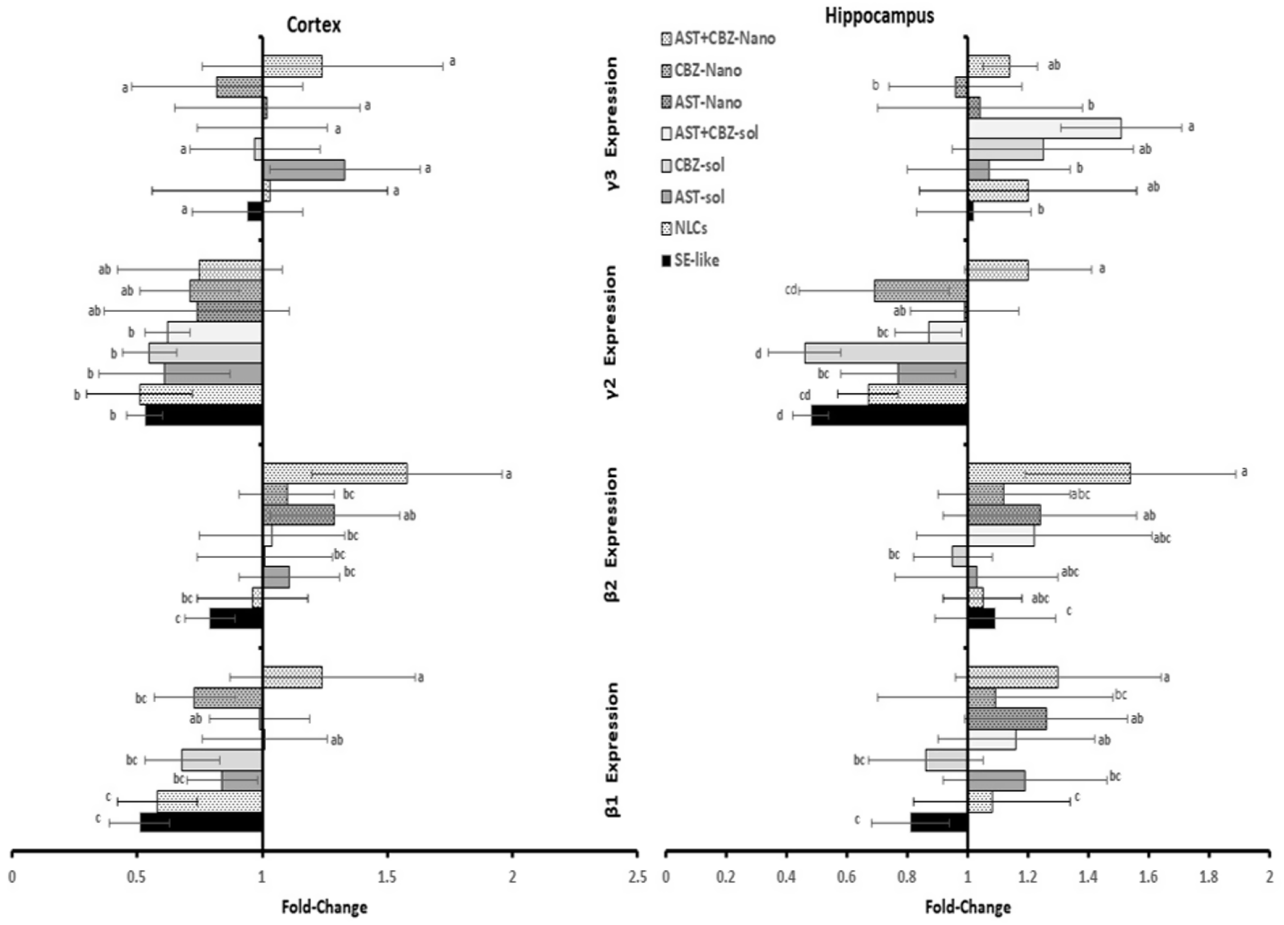
Results of the cortical and hippocampal expression of GABA receptor A (GABA_A_) β1, β2, γ2, and γ3 subunits of the studied groups. Data presented as Mean±SD and *n* = 8. Means with common letters are not significantly different, and means with different letters are significantly different by ANOVA followed by Tukey *post hoc*-test, *p* < 0.05. AST, astaxanthin; CBZ, carbamazepine; Nano, nanoparticles; NLCs, nano-lipid carriers (vehicle); SE, status epilepticus; sol, solution.

The SE-like rats showed significant suppression in the cortical gene expression of GABA_A_ receptor subunits β1 and γ2 while exhibiting suppressed expression of γ2 in the hippocampal tissues (Figure 4). The NLC-formulation treatments generally ameliorate derangements more effectively than solutions, especially the combined AST + CBZ-NLC, which normalized suppressed subunits (β1, γ2) in both cortex and hippocampus (Figure 4).

### 3.4 Serotonin and dopamine levels

Neurotransmitters, serotonin and dopamine (DA), showed a significant decline in both the cortex and the hippocampus. Treatment with AST nano-formulations is associated with a substantial increase in cortical serotonin and hippocampal dopamine (DA). The nanoformulations are more effective than the solutions, and the AST is more effective than CBZ in correcting serotonin levels in the cortex and DA levels in the hippocampus (Table 5).

### 3.5 Gephyrin expression in the cortex and hippocampus

**Figure 5:**
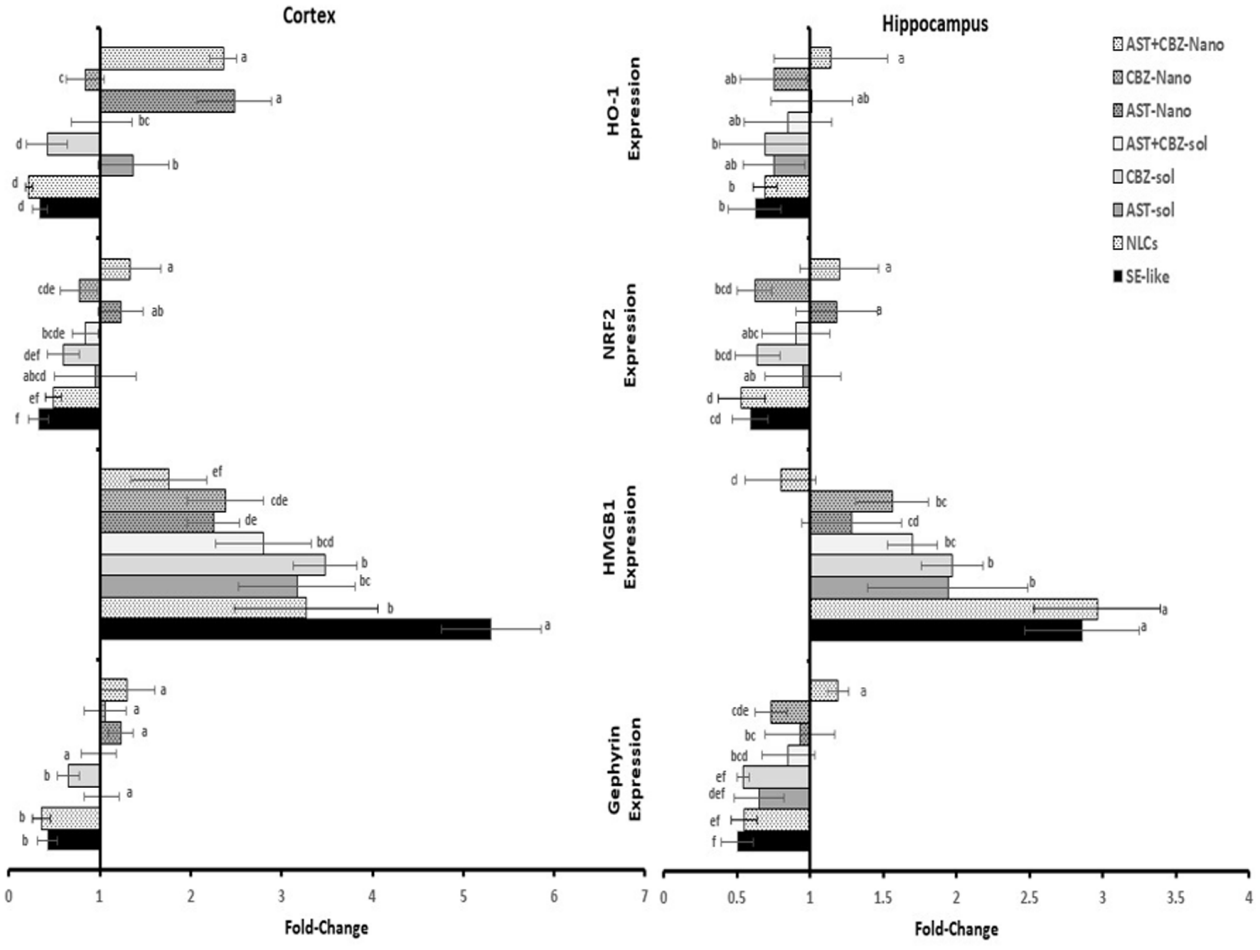
Results of cortical and hippocampal expression of gephyrin, HMGB1, NRF2, and HO-1 of the studied groups. Data presented as Mean±SD and *n* = 8. Means with common letters are not significantly different, and means with different letters are significantly different by ANOVA followed by Tukey *post-hoc* test, *p* < 0.05. AST, astaxanthin; CBZ, carbamazepine; Nano, nanoparticles; NLCs, nanolipid carriers (vehicle); SE, status epilepticus; sol, solution.

Gephyrin expression was significantly suppressed in the cortex and hippocampus of SE-like rats. Intranasal treatments with AST (solutions or NLCs, alone or combined) significantly corrected this suppression. CBZ treatments alone were found to be considerably less efficient than AST in restoring gephyrin expression (Figure 5).

### 3.6 Neuroinflammatory markers

**Figure 6:**
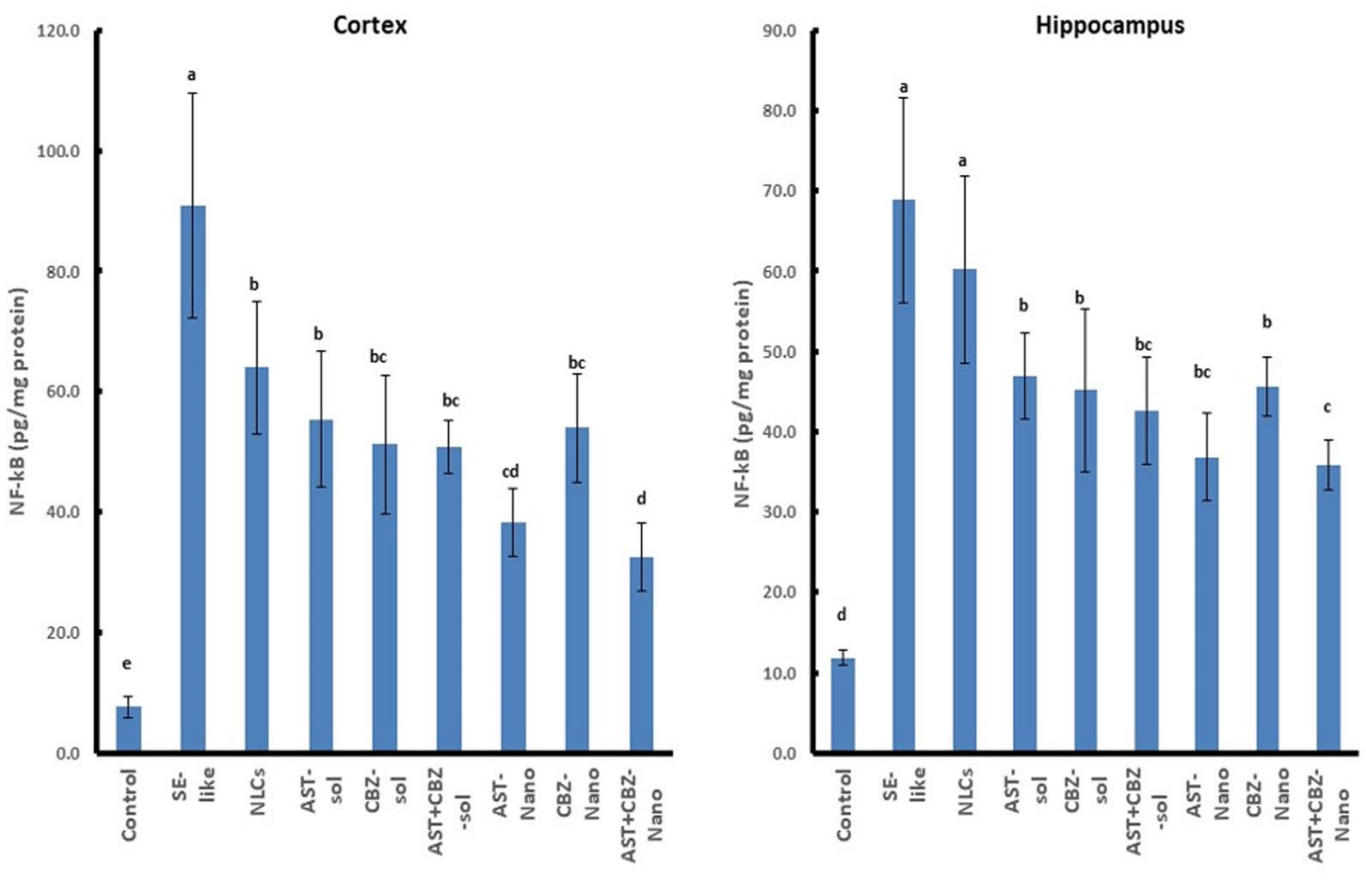
Results of cortical and hippocampal levels of NF-κB protein of the studied groups. Data presented as Mean±SD and *n* = 8. Means with common letters are not significantly different, and means with different letters are significantly different by ANOVA followed by Tukey *post hoc* test, *p* < 0.05. AST, astaxanthin; CBZ, carbamazepine; Nano, nanoparticles; NLCs, nanolipid carriers (vehicle); NRF2, nuclear factor erythroid 2 related factor 2; SE, status epilepticus; sol, solution.

Neuroinflammation was evident in SE-like rats, as indicated by a marked induction of HMGB1 expression (Figure 5) and significantly elevated NF-κB levels (Figure 6) in both the cortex and hippocampus. Treatment with AST alone or in combination with CBZ (solutions or NLCs) resulted in a significant decline in HMGB1 expression and NF-κB levels. CBZ therapies alone significantly decreased the expression of HMGB1 and NF-κB levels in the cortex and hippocampus, but to a lesser extent than the AST treatments (Figures 5, 6).

### 3.7 NRF2 antioxidant system in cortex and hippocampus

**Figure 7:**
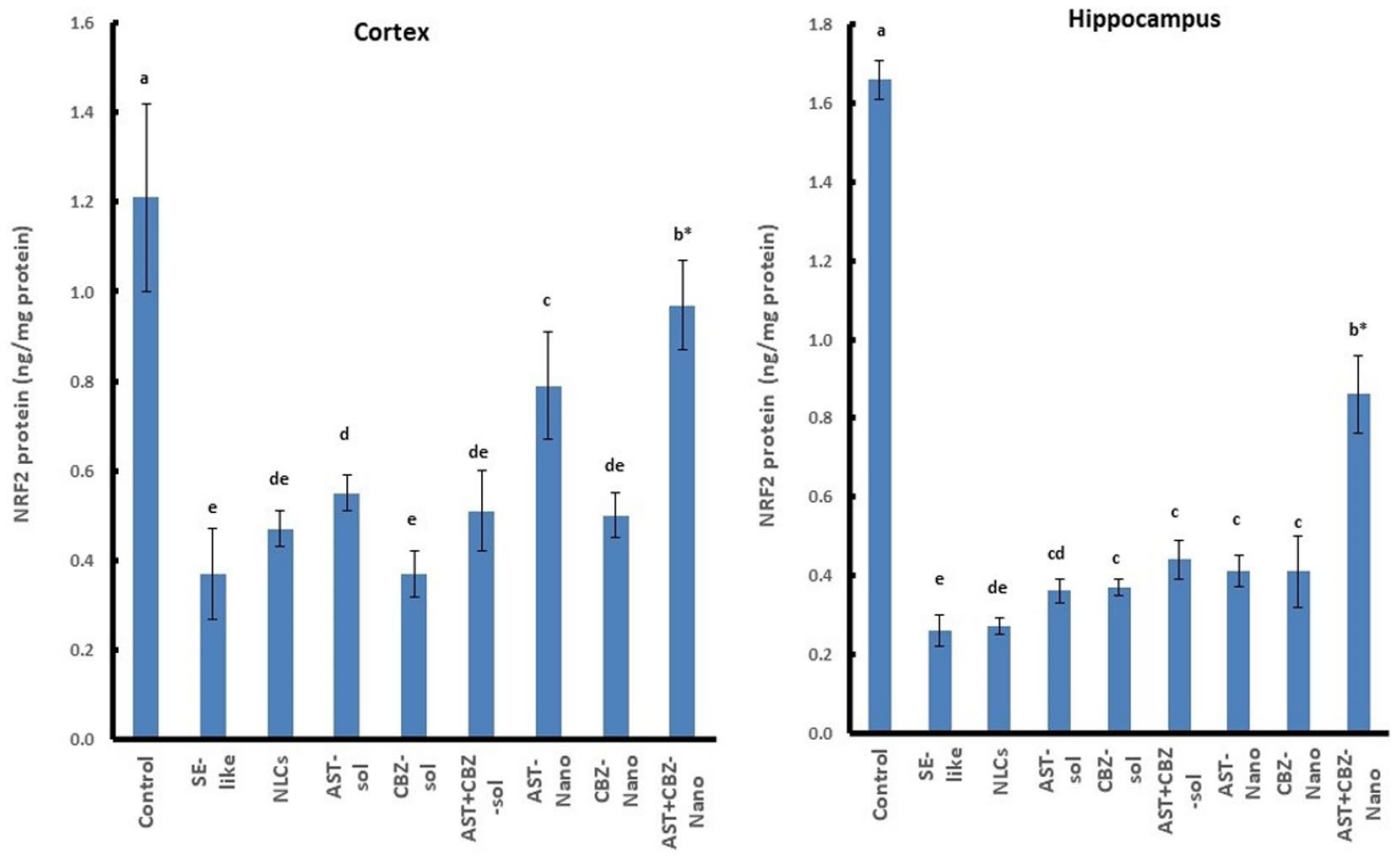
Results of cortical and hippocampal levels of NRF2 protein of the studied groups. Data presented as Mean±SD and *n* = 8. Means with common letters are not significantly different, and means with different letters are significantly different by ANOVA followed by Tukey *post-hoc* test, *p* < 0.05. *Synergistic interactions between AST and CBZ in the combined group using factorial design. AST, astaxanthin; CBZ, carbamazepine; Nano, nanoparticles; NLCs, nanolipid carriers (vehicle); NRF2, nuclear factor erythroid 2 related factor 2; SE, status epilepticus, sol, solution.

SE-like rats exhibited a significant decline in cortical NRF2 protein and mRNA (Figure 7), as well as HO-1 mRNA (Figure 5) in the cortex and hippocampus. AST solutions showed mild increases, while AST NLC formulations (alone or combined) significantly and completely normalized NRF2 and HO-1 expression in the cortex. CBZ-NLC only mild effects (Figures 5, 7). In the hippocampus, only AST preparations significantly upregulated and normalized NRF2 mRNA expression (Figure 7). HO-1 expression remained relatively unchanged, though the highest levels were seen with AST NLCs (Figure 5). For hippocampal NRF2 protein, only the combined AST + CBZ-NLC significantly and completely normalized its level (Figure 7).

### 3.8 Histopathological results and quantitative assessment of the number of degenerated and necrotic neurons

#### 3.8.1 In cortex

**Figure 8:**
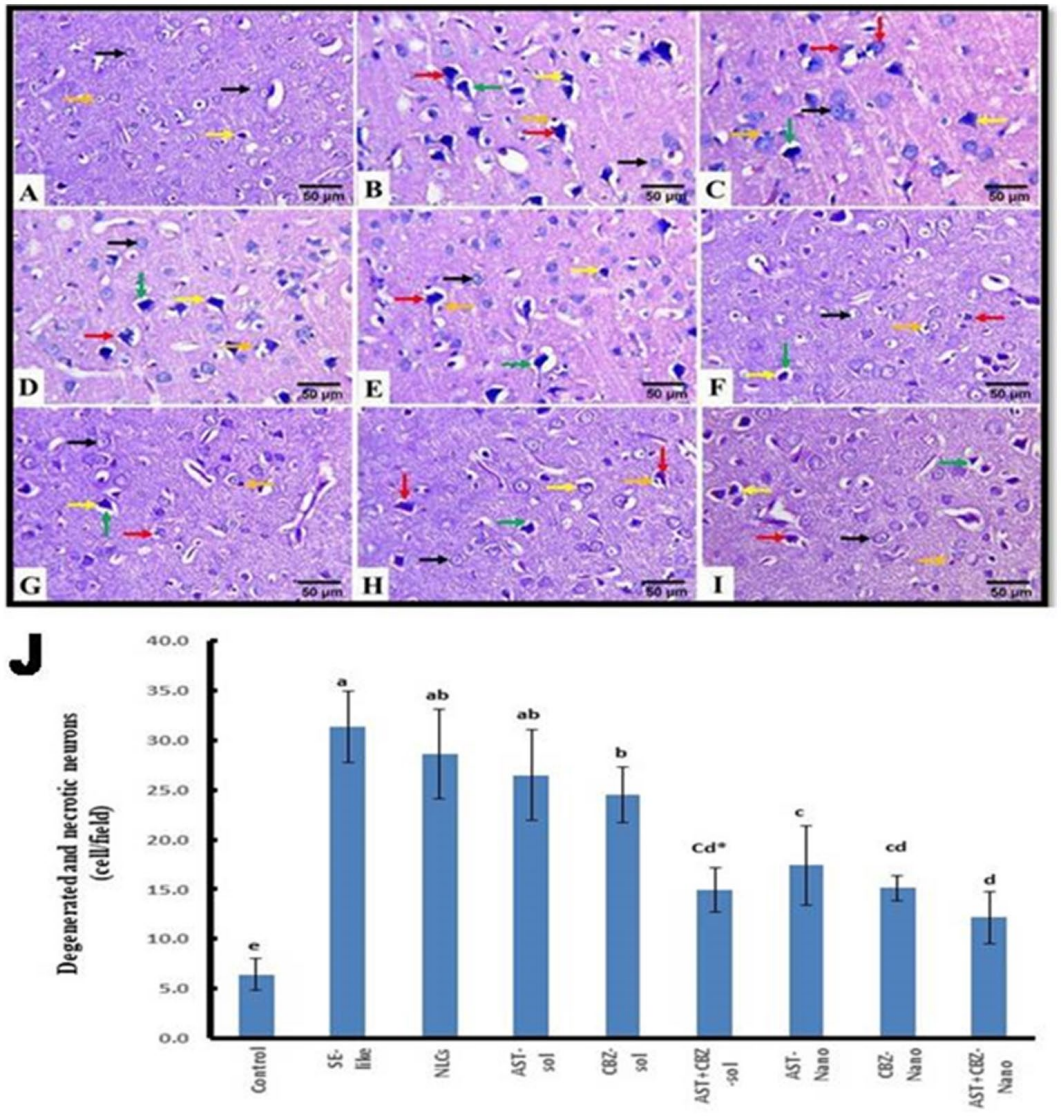
Representative photomicrograph of rat cerebral cortex (HE, ×400). **(A)** Control and **(B)** SE-Like, **(C)** NLCs, **(D)** AST-Sol, **(E)** CBZ-Sol, **(F)** AST + CBZ-Sol, **(G)** AST-Nano, **(H)** CBZ-Nano, and **(I)** AST + CBZ-Nano treated rats. Normal neurons (black arrow), degenerated neurons (yellow arrow), necrotic neurons associated with satellitosis and neuronophagia (red arrows), glial cells (orange arrow), and perineural space (green arrow). **(J)** Quantitative analysis of the number of degenerated and necrotic neurons in the cerebral cortex of the studied groups using Image J software. Data presented as Mean ± SD and *n* = 8. Means with common letters are not significantly different, and means with different letters are significantly different by ANOVA followed by Tukey *post-hoc* test, *p* < 0.05. AST, astaxanthin, CBZ, carbamazepine; Nano, nanoparticles; NLCs, nanolipid carriers (vehicle); NRF2, nuclear factor erythroid 2 related factor 2; SE, status epilepticus, sol, solution.

The histopathological findings of the brain cortical tissues of the different studied groups and the quantitative evaluation of the number of degenerated and necrotic neurons are represented in Figure 8. The control rats exhibited nearly normal histological structures of the cerebrum cortices, with a normal architecture of the neurons and neuropil (Figure 8A). The cerebral cortex of SE-like rats, on the other hand, displayed smaller and more deeply pigmented neurons with more perineural space. Additionally, there were visible necrotic neurons with pyknotic nuclei, either with or without satellitosis and neuronophagia (Figure 8B). This group showed a significant increase in the mean number of degenerated and necrotic neurons as compared with the control group (Figure 8J). Similar histological alterations were also detected in the NLCs group (Figure 8C), which showed a non-significant decrease in the mean number of degenerated and necrotic neurons compared with the SE-like rats (Figure 8J). In contrast, individual treatments of SE-like rats with AST (Figure 8D), CBZ (Figure 8E) or their combination (Figure 8F) as solutions and with AST (Figure 8G), CBZ (Figure 8H) or their combination (Figure 8I) as nanoformulations ameliorated the previous alterations and resulted in substantial reductions in the mean number of degenerated and necrotic neurons to various extents compared as compared with the in SE-like rats, however, they were comparable with the control rats (Figure 8J). The best modulatory effects were observed in the combined nanoformulations of AST and CBZ. followed by their combined solution form, then the CBZ loaded on NLCs (Figure 8J).

#### 3.8.2 The hippocampus

**Figure 9:**
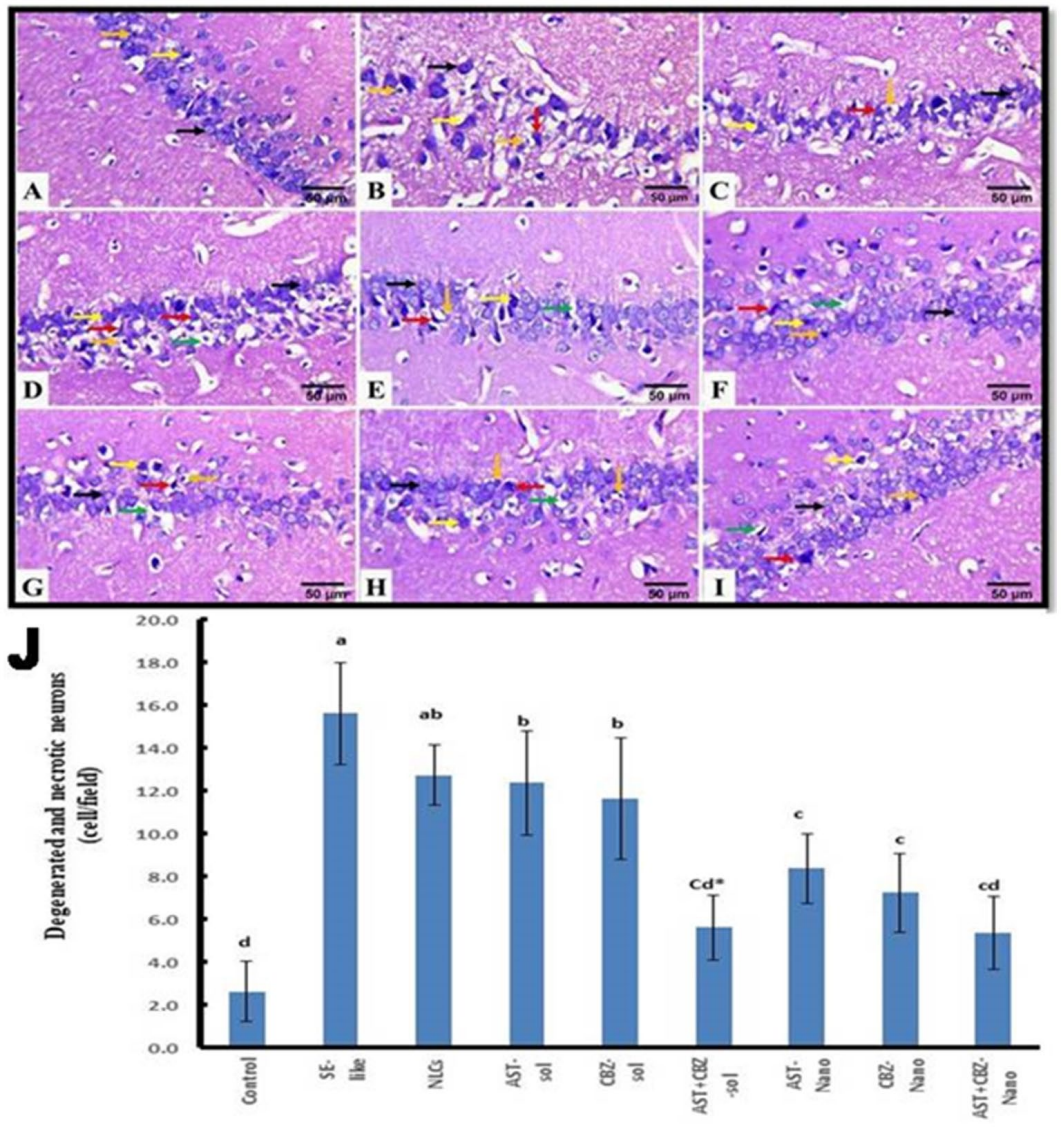
Representative photomicrograph of rat hippocampus (CA1, HE, 400×). **(A)** Control and **(B)** SE-Like, **(C)** NLCs, **(D)** AST-Sol, **(E)** CBZ-Sol, **(F)** AST + CBZ-Sol, **(G)** AST-Nano, **(H)** CBZ-Nano, and **(I)** AST + CBZ-Nano. Normal neurons (black arrow), degenerated neurons (yellow arrow), necrotic neurons associated with satellitosis and neuronophagia (red arrows), glial cells (orange arrow) and perineural space (green arrow). **(J)** Quantitative analysis of the number of degenerated and necrotic neurons (cell/field) in hippocampus (CA1) of the studied groups using image J software. Data presented as Mean ± SD and *n* = 8. Means with common letters are not significantly differ and means with different letters are significantly differ by ANOVA followed by Tukey *post-hoc* test, *p* < 0.05. *Synergistic interactions between AST and CBZ in the combined group using factorial design. AST, astaxanthin; CBZ, carbamazepine; Nano, nanoparticles; NLCs, nanolipid carriers (vehicle); NRF2, nuclear factor erythroid 2 related factor 2; SE, status epilepticus; sol: solution.

The histopathological findings of the hippocampal tissue, Cornu ammonis 1 (CA1) sub-region of the hippocampus, and the quantitative assessment of the number of degenerated and necrotic neurons are illustrated in Figure 9. The hippocampal tissue of the control rats showed normal morphology of the nerve cells (Figures 9A,J). However, in SE-like rats (Figure 9B), hippocampal tissues revealed malformed cellular morphology. Irregular and less cohesive neurons with a lot of space in between were detected. Additionally, degenerated and necrotic neurons were observed. This group showed a significant increase in the mean number of degenerated and necrotic neurons as compared with the control group (Figure 9J). Similar histological alterations were also detected in the NLCs group (Figure 9C), which showed a non-significant decrease in the mean number of degenerated and necrotic neurons as compared with the SE-like rats. Conversely, individual treatments of SE-like rats with AST (Figure 9D), CBZ (Figure 9E) or their combination (Figure 9F) as solutions and with AST (Figure 9G), CBZ (Figure 9H) or their combination (Figure 9I) as nanoformulations ameliorated the previous alterations and to various extents resulted in significant decrease in the mean number of degenerated and necrotic neurons as compared with the SE-like rats (Figure 9J). However, they were comparable to the control rats. When administered in nanoformulation, AST or CBZ significantly reduced the number of degenerated and necrotic neurons in SE-like rats compared to when administered in solution form. Treatment with either AST or CBZ as a nanoformulation significantly reduced the number of cells in SE-like rats compared to treatment with their solution forms. Compared to AST or CBZ alone (as a solution or nanoformulation), the combination therapies (as a solution or nanoformulation) had a greater impact on reducing the mean number of degenerated and necrotic neurons. Rats treated with the combined nanoformulation showed the most significant reduction in the number of degenerated and necrotic neurons (Figure 9J).

### 3.9 Correlation studies

The Pearson correlation coefficient between different parameters in the cortex and hippocampus is presented in Table 6.

**Table 6:** Correlation coefficient between different parameters in the cortex and hippocampus of SE-like rats.

Parameters	Tissue	HO-1 expression	NRF2 expression	Dopamine	Serotonin	NF-ĸB	NRF2 protein	GABA	Degenerated necrotic neurons
HMGB1	Cortex	−0.630*	−0.552*	−0.600*	−0.525*	0.763*	−0.641*	−0.527*	0.714*
	Hippocampus	−0.426*	−0.673*	−0.559*	−0.552*	0.729*	−0.722*	−0.570^*^	0.673*
Gypherin	Cortex	0.722*	0.704*	0.513*	0.578*	−0.623*	0.633*	0.676*	−0.682*
	Hippocampus	0.618*	0.548*	0.412*	0.375*	−0.488*	0.759*	0.544*	−0.627*
GABA_A_β1	Cortex	0.637*	0.691*	0.330*	0.341*	−0.546*	0.684*	0.605*	−0.619*
	Hippocampus	0.341*	0.316*			−0.254*	0.339*		−0.300*
GABA_A_β2	Cortex	0.602*	0.620*	0.392*	0.359*	−0.500*	0.665*	0.587*	−0.498*
	Hippocampus		0.251*		0.313*	−0.255*	0.554*	0.457*	−0.379*
GABA_A_α1	Cortex	0.533*	0.675*	0.477*	0.543*	−0.547*	0.569*	0.694*	−0.603*
	Hippocampus	0.515*	0.452*	0.265*	0.360*	−0.483*	0.613*	0.475*	−0.622*
GABA_A_α2	Cortex	0.270*	0.423*	0.247*	0.404*	−0.296*		0.400*	−0.257*
	Hippocampus	0.461*	0.414*	0.306*			0.389*	0.326*	−0.393*
GABAAα5	Cortex	0.645*	0.755*	0.465*	0.659*	−0.463*	0.704*	0.550*	−0.527*
	Hippocampus		−0.322*	−0.343*	−0.513*	0.536*	−0.338*	−0.367*	0.486*
GABA_A_γ2	Cortex	0.385*	0.305*	0.309*	0.401*		0.409*		−0.298*
	Hippocampus	0.509*	0.519*	0.444*	0.295*	−0.488*	0.668	0.434*	−0.536
HO-1 expression	Cortex		0.746*	0.398*	0.732*	−0.646*	0.863*	0.665*	−0.608
	Hippocampus		0.454*			−0.270*	0.408*	0.291*	−0.313*
*NRF2 expression*	Cortex			0.367*	0.664*	−0.599*	0.755*	0.679*	−0.578*
	Hippocampus			0.506*	0.304*	−0.531*	0.486*	0.498*	−0.432*
NRF2 Protein	Cortex							0.662*	−0.583*
	Hippocampus							0.535*	−0.629

*Significant correlation at *p* ≤ 0.05.

## 4 Discussion

This study provides compelling evidence for the anti-epileptic potential of intranasal astaxanthin (AST), either alone or in combination with carbamazepine (CBZ), particularly in nanostructured lipid carrier (NLC) formulations, in a LiCl-pilocarpine-induced rat model of status epilepticus (SE). The findings demonstrate that AST and AST + CBZ-NLC formulations significantly ameliorate behavioral deficits (impaired motor coordination and spatial memory deficits), reduce neuronal degeneration, and restore biochemical and molecular parameters in the cortex and hippocampus, with the combined NLC formulation showing superior efficacy.

The intranasal doses of AST at 4 mg/kg and CBZ at 0.2 mg/kg used in the present study were selected based on prior preclinical studies demonstrating efficacy and safety in rodent models of neurological disorders, including epilepsy and related conditions. The AST dose was supported by studies showing neuroprotection and cognitive benefits of the same dose (Deng et al., 2019; Bhatt et al., 2016), with the intranasal route chosen to enhance direct brain delivery by bypassing the blood–brain barrier, a strategy validated by nanostructured lipid carrier (NLC) studies (Shehata et al., 2023). So, the used AST dose (4 mg/kg) was deemed optimal for achieving therapeutic efficiency in the brain while minimizing systemic exposure. The CBZ dose of 0.2 mg/kg was selected based on intranasal studies showing effective seizure reduction in rats at low doses, with minimal systemic side effects compared to oral routes (Barakat et al., 2006), which aligns with the potential for enhanced brain targeting via this route. NLC formulations were used to improve the solubility, stability, and brain delivery of both lipophilic drugs (Elmowafy et al., 2018). The combination was tested to explore synergistic effects, maintaining monotherapy doses due to a lack of established combination data and funding constraints, yet it still yielded significant therapeutic outcomes.

Translating the used doses to humans involves calculating Human Equivalent Doses (HEDs) using body surface area normalization (Department of Health and Human Services, United States, Food and Drug Administration, Center for Drug Evaluation and Research, 2005). The 4 mg/kg AST dose translates to 0.648 mg/kg HED (approximately 45.36 mg/day for a 70 kg human), within the range of safe oral doses used clinically (Ambati et al., 2014). However, the intranasal route may require lower doses due to enhanced brain bioavailability. Studies on intranasal drug delivery suggest that 10–20% of the oral dose may suffice for equivalent brain concentrations due to direct nose-to-brain transport (Costa et al., 2021). Therefore, an intranasal AST dose of 5–10 mg/day in humans may be sufficient, which warrants further pharmacokinetic studies. However, the translatability to human dosing faces challenges, such as species differences in nasal physiology and the need for human trials to confirm safety, tolerability, pharmacokinetics, and optimal dosing for intranasal AST and CBZ, especially in combination, despite promising preclinical synergistic effects.

The pilocarpine SE model replicates key features of human temporal lobe epilepsy, including seizures, hippocampal neuronal loss, and cognitive/behavioral alterations (Delgado-Escueta et al., 1980; Lee et al., 2018). SE-like rats exhibited impairments in motor coordination and spatial learning/memory (Müller et al., 2009), linked to hippocampal damage. Treatment interventions, particularly NLC formulations (CBZ alone or combined with AST), significantly improved behavioral performance, highlighting the nanodelivery advantage. AST treatments yielded comparable cognitive benefits, likely mediated through their antioxidant and anti-inflammatory properties, offering neuroprotection.

At the histopathological level, the examination revealed significant neuronal damage (degeneration, necrosis, pyknotic nuclei) within the cortex and hippocampus of SE-like rats (Kim and Lee, 2016; Grote et al., 2022). These changes were substantially ameliorated by therapeutic interventions, especially NLC formulations, which provided superior protection compared to solutions, reinforcing the value of nanoformulation in preserving neuronal integrity. While combined solutions showed synergy, NLC monotherapies often offered better protection than solution forms.

These histological improvements correlated with positive modulations within critical neurotransmitter systems. SE-like rats exhibited reduced GABA levels in the cortex and hippocampus, aligning with human epilepsy observations (Gonen et al., 2020; He et al., 2021), and potentially linked to the pathogenesis of epilepsy (Kakizaki et al., 2021). Serotonin and dopamine levels were also decreased, alterations implicated in epileptogenesis (Bozzi and Borrelli, 2013). Dopamine has anti-convulsant properties (Starr, 1996; Clinckers et al., 2005), reduced levels are seen in epilepsy (Alcantara-Gonzalez et al., 2013; Rocha et al., 2012). Combined treatments (solutions or nano) effectively restored GABA, serotonin, and dopamine levels. Nanomonotherapies demonstrated partial correction, supporting the use of combination therapy and nanodelivery in rebalancing neurotransmission.

Due to the crucial role of GABA receptors in regulating GABAergic neurotransmission, neuronal excitability, and seizure activity, we investigated the GABAergic system, focusing on the expression of the essential subunits of GABA_A_ receptors (α1, α2, and α5; β1 and β2; and γ2 and γ3). SE-like rats exhibited complex alterations, as evidenced by the suppression of cortical expression of α1, α5, β1, and γ2. In contrast, in the hippocampus, the expression of α1, α2, and γ2 was suppressed, and expression. In contrast, in the hippocampus, the expression of α1, α2, and γ2 was suppressed. Expression of α5 induced, likely disrupting the balance between synaptic (phasic) and extrasynaptic (tonic) inhibition, contributing to hyperexcitability (Fritschy, 2008; Fritschy et al., 1995; Houser et al., 2012; Hines et al., 2012). For instance, suppressed cortical α1, β1, and γ2 subunits could impair phasic inhibition, while hippocampal changes might affect both phasic and tonic inhibition (Houser et al., 2012). NLC formulations, particularly the AST + CBZ combination, demonstrated the most consistent efficacy in normalizing the expression of several critical subunits (e.g., α1, β1, β2, and γ2 in the hippocampus), suggesting a significant restoration of GABAergic function. However, proper receptor assembly and localization (both synaptic and extrasynaptic) are also essential (Sente et al., 2022).

Gephyrin, a crucial scaffolding protein for clustering and stabilizing GABA_A_ receptors (Pizzarelli et al., 2020), was significantly suppressed in SE-like rats, potentially impairing receptor stability and function (Zhou et al., 2019). AST treatments (solution/nano, mono/combined) and the combined NLC formulation effectively restored gephyrin expression, particularly in the hippocampus, suggesting a mechanism for enhancing inhibitory synaptic function and stability.

Neuroinflammation, a key contributor to epilepsy pathogenesis (Turrin and Rivest, 2004), was evident via elevated NF-κB levels and induced HMGB1 expression. HMGB1 promotes inflammation via TLR4/NF-κB signaling (Kaneko et al., 2017) and targeting it shows therapeutic promise. All tested treatments significantly reduced these inflammatory markers. Generally, NLC formulations exhibited superior anti-inflammatory effects compared to solutions, and AST appeared more potent than CBZ, suggesting that AST directly counteracts inflammatory pathways in SE.

Oxidative stress, associated with neuroinflammation, involves dysregulation of the NRF2 antioxidant pathway (Wang et al., 2014). In the present study, NRF2/HO-1 levels were found to be negatively correlated with neuronal degeneration in SE-like rats. AST treatments, particularly NLC formulations, significantly restored NRF2 and HO-1 expression, boosting intrinsic antioxidant defenses. This aligns with AST’s antioxidant properties (Lu et al., 2015), and the protective role of the NRF2/HO-1 pathway (Ambati et al., 2014; Ji et al., 2017). HO-1 activity also inhibits NF-κB, contributing to anti-inflammatory effects (Ahmed et al., 2017). Correlation studies substantiated the interplay between antioxidant defenses, GABAergic function, neurotransmitters, inflammation, and neuronal damage.

## 5 Conclusion

This study provides robust support for the anti-epileptic potential of intranasal AST, particularly combined with CBZ and formulated as NLCs. The therapeutic benefits stem from a multifaceted mechanism involving the modulation GABAergic neurotransmission (receptor subunits and gephyrin), the restoration of serotonin and dopamine, the alleviation of neuroinflammation (NF-κB and HMGB1), and the enhancement of endogenous antioxidant defenses (NRF2/HO-1). The consistent superiority of NLC formulations highlights the advantages of this delivery system in improving brain targeting and efficacy. Synergistic effects with AST + CBZ-NLC therapy suggest its potential as a promising novel strategy for managing epilepsy’s complex pathophysiology.

The clinical implications of these findings are substantial. Intranasal delivery of AST and CBZ, particularly in NLC formulations, offers a promising strategy to enhance brain targeting, improve bioavailability, and minimize systemic side effects compared to conventional oral AED therapies. The safety profile of AST, a natural antioxidant with established use in other neurological disorders, further supports its potential as an adjunctive therapy to enhance the efficacy of CBZ while mitigating epilepsy-induced neural damage. However, successful clinical translation requires further research, including dose optimization, pharmacokinetic assessments (both in animals and humans), deeper mechanistic investigations (such as electroencephalogram [EEG] and protein analysis), and validation in diverse epilepsy models. Ultimately, well-designed clinical trials are indispensable to confirm the safety, tolerability, and efficacy of this novel intranasal co-therapy approach for improving seizure control and quality of life for patients with epilepsy.

## Data Availability

The original contributions presented in the study are included in the article/supplementary material, further inquiries can be directed to the corresponding author/s.

## Glossary

%EE: Entrapment efficiency percent
AEDs: Antiepileptic drugs
AST: Astaxanthin
b.wt: Body weight
BBB: Blood–brain barrier
CBZ: Carbamazepine
cDNA: Complementary deoxyribonucleic acid
CNS: Central Nervous System
Ct: Threshold cycle
DA: Dopamine
DNA: Deoxyribonucleic acid
dNTPs: Deoxyribonucleotides triphosphate
dT: Deoxythymine
dTTP: Deoxythymidine 5′-triphosphate
ELISA: Enzyme-linked immunosorbent assay
GABA: Gamma-aminobutyric acid
GABA_A_Rs: Gamma-aminobutyric acid type-A receptors
H&E: Hematoxylin and eosin
HMGB1: High mobility group box 1
HO-1: Heme oxygenase-1
IN: Intranasal
LiCl: Lithium chloride
mRNA: Messenger ribonucleic acid
MWM: Morris water maze
NF-κB: Nuclear factor kappa-light-chain-enhancer of activated B cells
NLCs: Nanostructured lipid carriers
NPs: Nanoparticles
Nrf-2: Nuclear factor transcription factor E2-related factor 2
PDI: Polydispersity index
PS: Particle size
qRT-PCR: Quantitative real-time polymerase chain reaction
RNA: Ribonucleic acid
ROS: Reactive oxygen species
SE: Status epilepticus
ZP: Zeta potential
